# Medical and Physical Intelligence

**Published:** 1820-09

**Authors:** 


### Medical and Physical Intelligence.

TRANSACTIONS OF SCIENTIFIC SOCIETIES.

ROYAL SOCIETY *of London*.—Dr. Wollaston has been elected President of the Society, *ad interim*.

*June* 29.—A paper by Dr. Wollaston was read, entitled "*On Sounds inaudible by certain Ears.*" The author, after some introductory remarks, proceeded to describe insensibility to certain sounds in persons not otherwise deaf, which he was led to observe by trying different modes of diminishing the sense of hearing in himself. He found, for example, that when the membrana tympani was thrown into a state of tension by external pressure, by closing the nose and mouth and expanding the chest, the ear was rendered insensible to grave tones, while its sensibility to sharper sounds was not affected. In this case all sounds below F in the bass cliff were inaudible.

In the healthy state of the ear, the power of discerning low sounds is great, and of uncertain limits; but, if we attend to the opposite extremity of the scale of audible sounds, and, with a series of small pipes succeeding each other in sharpness, observe their effects successively upon the ears of different persons, we shall find considerable difference in their powers of hearing them, and see reason to infer that the sense of hearing in man is more limited than has been supposed. The author's attention was particularly called to this circumstance, by observing a person insensible to the sound of a small organ-pipe, which, with respect to acuteness, was far within the limits of his own hearing. By subsequent examination, it was found that this person's hearing terminated at a note four octaves above the middle E of the piano-forte. Other instances were then referred to of the insensibility of certain persons to various acute sounds; such as the chirping of the grashopper, cricket, and sparrow, and especially the squeak of the bat, the existence of which is unknown to many individuals, from its being inaudible to them. The pitch of this sound was stated to be about five octaves above the middle E before mentioned. The author fixed the limits of his own hearing at six octaves above the same note; and stated, that, from numerous trials, he was induced to think that the interval of a single note between two sounds was sufficient to render the higher note inaudible.

The range of human hearing includes upwards of nine octaves, the whole of which are distinctly audible to most ears, although the vibrations of the acuter sounds are 600 or 700 times more frequent than those of the lower; and the author concluded by observing, that it is very probable that other animals are so organized as to be able to distinguish sounds still more acute, and of course inaudible by human ears, and thus to possess what may be considered as a new sense.

A very useful *Toxicological Chart** has been recently published, which is well worthy of the attention of the profession in general. It occupies one of the surfaces of two sheets of paper, and contains, arranged in respective columns, the names of the poisons, the symptoms of their effects, the treatment of them, and their proper tests; the whole of which are stated, as it professes, according to the latest experiments and observations. The design of the thing is good, and it is effected in a manner deserving of unqualified approbation. There is hardly any medical practitioner who might not, occasionally, find this chart useful to him, by refreshing his memory on more or less interesting points in cases of sudden emergency; and to those who have pupils liable to be called to cases of this kind in their absence, which happens commonly in the country, we earnestly recommend it, for the purpose of being stuck up in their surgery or dispensary. By being thus almost constantly in the view of a pupil, he would soon become tolerably well versed in the more immediately practical part of toxicology, and could thus at any time know, in one minute, what to do in the ordinary cases of poisoning.

---

The following observations, selected from an account given by Dr. Hamel, physician to the Emperor of Russia, of his descent in the sea in a diving-bell, seem to be worthy of the attention of the medical practitioner. When he had arrived at about four or five feet below the surface of the water, he began to feel pain in his ears, which became more and more severe as he descended. He began to fear that it would become quite intolerable, and made some efforts to introduce air into the Eustachian tubes, to counterbalance the inordinate pressure of that externally against the membrane of the tympanum. It was a long time before he could succeed in this, and then only with the right ear: on the air suddenly entering in this, the pain instantly ceased; but it became more and more distressing in the other ear. When he was at the depth of fifteen or sixteen feet, it seemed as if a stick were forcibly thrust into this ear. At length he made the air pass also on this side, which it did with a remarkable explosion, and the pain suddenly ceased. Not the least inconvenience was felt in respiration, even at a much greater depth; though the pressure of the atmosphere must have very considerably increased. On rising, he experienced again pain in his ears, resulting from the dilatation of the air in their interior cavities; but its escape was much easierthan its entry. He felt, at about each foot of distance, as it were a bubble of air escape from the ear into the mouth, and each time this took place the uneasiness lessened. As the orifice of the Eustachian tube is closed in the manner of a flattened tunnel towards the mouth, which acts like a valve, it is very difficult to make the air pass from the ear

* *A Toxicological Chart: in which is exhibited at one view the Symptoms, Treatment, and Modes of Detecting, the various Poisons, Mineral, Vegetable, and Animal; according to the latest Experiments and Observations.* By a Member of the Royal College of Surgeons in London. Published by Anderson, Medical Bookseller, 40, West Smithfield.

into the mouth under the ordinary atmospherical pressure; but, in the diving-bell under water, the action of deglutition alone is sufficient: it seems as if the muscles of the cheeks during this action opened the orifice of the tube, and that the condensed air then forced the passage. In making this experiment, it is necessary to close the nostrils, and to make then a strong suction with the mouth closed; the air then comes from the internal cavity of the ear into the mouth, and a slight degree of pain is felt; which ceases on swallowing the saliva; because the air then re-enters the Eustachian tube, and the equilibrium is again established. From these circumstances, Dr. Hamel thinks that the diving-bell might prove a remedy in cases of deafness from obstruction of the Eustachian tube. In the conversation he had with the workmen who ordinarily went down in it, he found that they experienced similar sensations in their ears; and one of them said that, when the pain had become very violent, he sometimes heard a noise like the sound of a pistol-shot, and then it instantly ceased.

Lady Hardy, and some other persons, who went down in the bell at Plymouth, stated that they experienced similar phenomena.

---

Dr. Mongeny, physician to the hospital of the colony Fernandina, in the isle of Cuba, states that he has discovered a new remedy for tape-worm, of extraordinary efficacy. It consists in giving to the patient, fasting, three ounces of a paste made of fresh *citrouille*,* and afterwards six ounces of honey in three doses; the first at the end of an hour, and the others after similar intervals. By this process, Dr. M. says, the tænia has always been expelled after the lapse of six or seven hours; and in many of these cases all the medicines of most reputed efficacy had been tried without success. The worm is voided knotted and rolled up in a ball, and not in fragments, as is commonly the case when it is expelled by other anthelminthics. In subjects who had two of these worms, they were both expelled at the same time, in the way just described.

---

Dr. Perret has communicated to the Physical Society of Carcassonne, a series of observations in proof of the efficacy of the plantain-root in intermittent fevers. It has long been employed in such cases by the German physicians. As it is an indigenous plant in England, it is extremely desirable that it should be ascertained whether or not the virtues imputed to it are well-founded; especially since Humboldt found, in his last voyage in America, that the cinchona lancifoliata was becoming exhausted in the forests about Peru.

* We give the original, because we do not know what plant Dr. M. designates: neither does the Editor of the *Journal Universel des Sciences Medicales*, to whom the account of the remedy was sent in a letter; since he expresses a desire that Dr. Mongeny had described its botanical characters. By his not having done this, we are inclined to suppose that it is a well-known plant. Now *citronelle* is *balm*, (*melissa*, Lin.) it is possible that this may be meant, and that the MS. has not been so legibly written as to be properly comprehensible by the Editor of the Journal from whom we derive this account.

The following extraordinary and severe accidents from the bite of a leech lately occurred in a patient of Dr. Marquand, of Paris. Dr. M. had prescribed the application of some leeches below the ears of a man who had been burned just over the eyelids by a hot iron, and who had severe pain in the back part of his head, apparently in consequence of this accident.

The leeches were applied on the sides of the neck, and one of them fixed in the direction of the sterno-cleido-mastoid muscle, on the right side. The patient immediately suffered acute pain, which was instantly followed by involuntary contraction of that muscle, which turned his head to the left, and drew his right ear down on the shoulder. The contractions were so violent that the patient held his head with both his hands, to moderate the shocks of them. After this he suffered permanent contraction of the neck in this manner, accompanied with pains which extended from the right side of the neck to the chest of the same side. He was found in this state by Dr. Marquand, twenty-four hours after the application of the leeches; the bites of which did not, however, present any thing peculiar. The patient no longer suffered pain in his head, but severely from that just mentioned, which, with the fatigue from the severe contractions, kept him in a constant sweat. Dr. M. considered these symptoms to arise from puncture of a filament of the portio dura of the seventh pair of nerves, and not from the accident for which the leeches were applied. An opiate liniment was rubbed on the part affected, which relieved, in two hours, the contractions of the muscle in the neck; but they now extended further, and affected those of the back, so as to shake the patient in his chair. A quarter of a grain of opium every five hours, was ordered in addition to the liniment. The convulsive muscular contractions did not entirely cease until the lapse of a week; and, previously to their disappearance, they had shifted to the left lower extremity, the leg of which was now and then suddenly bent with violence upon the thigh. Three weeks after the accident he returned to his labour, as a whitesmith: the first day he suffered two or three spasms, which extended from the neck to the back and the left leg; but since that time he has been free from them.

---

*Antidote for Vegetable Poisons.*—M. Drapiez has ascertained, by numerous experiments, that the fruit of the *fewillea cordifolia* is a powerful antidote against vegetable poisons. This opinion has been long maintained by naturalists, but I am not aware that it has ever before been verified by experiments made on purpose in any part of Europe. M. Drapiez poisoned dogs with the rhus toxicodendron, hemlock, and nux vomica. All those that were left to the effects of the poison, died; but those to whom the fruit of the fewillea cordifolia was administered, recovered completely, after a short illness. To see whether this antidote would act in the same way, when applied externally to wounds into which vegetable poisons had been introduced, he took two arrows which had been dipped in the juice of manchenille, and slightly wounded with them two young cats. To one of these he applied a poultice, composed of the fruit of the fewillea cordifolia, while the other was left without any application. The former suffered no other inconvenience except from the wound, which speedily healed; while the other in a short time fell into convulsions and died.

It would appear from these experiments, that the opinion entertained of the virtues of this fruit in the countries where it is produced, is well founded. It would deserve in consequence to be introduced into our Pharmacopœias as an important medicine; but it is necessary to know that it loses its virtues, if kept longer than two years after it has been gathered.

---

*Prize Questions.*—A satisfactory answer not having been given to the question, "Can the existence of idiopathic fevers be doubted?" proposed last year by the Societé de Medecine of Paris, it is again proposed for a new concourse of candidates.

The Society also declares, that it will leave to the candidates the greatest latitude in the choice and development of their opinions.

The prize will consist in a gold medal of the value of 300 francs. But the Society, considering the importance of the subject, will attribute, besides, if there be opportunity, to the memoirs which have the most nearly attained the prize, gold medals of the value of 100 francs, and silver medals of emulation.

The concourse will be closed on the 30th of September, 1821. The memoirs, legibly written in French or in Latin, should arrive, carriage-free, before the above period, at the residence of the "Secretaire Général de la Societé de Medecine," rue Sainte-Avoir, No. 39.*

---

The *Academy of Sciences* of Paris proposes the following:

"To follow the development of the triton, or aquatic salamander, in its different degrees, from the egg to the perfect animal, and to describe the change which it undergoes interiorly, principally in respect to its osteology and the distribution of its vessels."

The prize, of the value of 300 francs, will be adjudged in the public sitting of 1822. The utmost term for the transmission of memoirs is the 1st of January, 1822.

---

Medical magnetism continues to extend its influence in various parts of Germany, and to count almost daily new additions to its votaries, even amongst physicians well instructed, and otherwise men of sobriety and good conduct. One of them, Dr. Gamard, of the Faculty of Halle in Prussia, has lately settled in Philadelphia, with the view of trying the fortune of this doctrine amongst the United-States-men. But, it seems that neither the physicians nor the people of this headstrong nation have treated him very kindly; for he has just sent his observations on the subject, in which he relates *only one case*, to the

* We think it proper to mention, that no distinction is made in regard to nation in the concurrents for the prize, because we never find the names of our countrymen amongst them.—Edit.

Society of the Faculty of Paris, and this case presents, too, a deplorable picture of the fanaticism, the *monomania*, of its author. It is that of a woman who had abdominal dropsy, which appeared to be encysted. Dr. Gamard first prescribed for her purgative pills, a decoction of water-cresses with nitre, whey with nitre in it, &c. under which treatment the dropsy disappeared; and the cure seemed to be nearly complete, when, omitting the medicines, the patient suffered a relapse. It was now that the Doctor began the use of galvanism, all the proceedings of which he describes with much minuteness; but, recollecting the benefit that had been derived from the medicines employed in the first instance, he resorted to them from time to time, and some slight alleviation was experienced from them; but, like the dog and the shadow, he neglected the reality for his favorite magnetism, to which he attributes every good. After a month's trial of these mixed measures, the patient became weary of them, ceased to use them, and died. This case is, nevertheless, brought forward by Dr. Garnard, as a proof of the efficacy of magnetism ; and he believes that it would have cured his patient, had she persevered in its use. The Faculty of Paris have thought differently, and have had the memoir deposited amongst their archives as a specimen of medical credulity.

---

A very interesting case of laceration of the subclavian artery by a shot, in which the hemorrhage was permanently restrained by compression, has been published by Dr. Bedeschi (in the *Annali Universali di Medicina*, Feb. 1820). A man, twenty-seven years of age, received a shot on the middle of the right scapula, which traversed the chest so as to fracture the clavicle in the middle and lacerate the subclavian artery and vein, and the adjacent nerves ; judging by the direction of the wound, and by the great loss of arterial blood which the patient had experienced. The right arm was cold, and devoid of sense and the power of motion. A compress was applied over the artery as it passes from between the scaleni muscles, and graduated compresses and a bandage applied over this. Warm fomentations were applied to the arm. Although three days elapsed without any serious consequences, some medical men advised the amputation of the arm, or the application of a ligature to the subclavian artery, to prevent more certainly a destructive hemorrhage on the separation of the eschar. But Dr. Bedeschi declined resorting to these measures, relying, 1st. on the suspension of the hemorrhage by the eschar, and the clot of coagulable lymph in the vessel; 2d. on the sufficient means presented for the compression of the artery at its passage from the scaleni on the first rib by the sternal portion of the clavicle, which the fracture had rendered movable; 3d. because this difficult and uncertain operation might be retarded; and, lastly, because fatal hemorrhages have not often been consequent on gun-shot wounds, and many severe wounds of arteries have been cured by compression. No hemorrhage troubled the course of the cure ; a little emaciation of the arm, and impossibility of moving it, were the only evil consequences. The pulsation of the subclavian artery was afterwards found to be, very strong for half an inch beyond the scaleni, but could hardly be felt in the axilla: it began to be perceptible again about the middle of the arm, and was very strong at the wrist, though the artery was a little contracted. Moderate exercise of the limb, and some other ordinary measures, restored its strength; so that the patient had recovered the use of it after the lapse of twenty-eight months from the accident.

---

*Mineralogy.*—M. Beudant continues to enrich crystallography with researches equally new and interesting. We saw last year, by his experiments, how a saline principle of a certain kind sometimes impressed its crystalline form upon a mixture in which it did not by any means form the greatest part.

He has occupied himself this year with a question that is not less important in respect to the knowledge of crystals; namely, to determine the causes which occasion a saline substance, whose primitive molecules and nucleus have a constant form, to be disguised, by means of the accumulation of its molecules according to different laws, with so many and so various secondary forms, that their number is sometimes astonishing.

Having remarked that the secondary forms of a substance are most commonly the same in any one mine or place where it is found associated with other minerals under similar circumstances, he judged that the secondary forms arise from the medium in which the crystallization takes place.

It has been known for a long time, from the experiments of Romé de Lille, and those of Fourcroy and M. Vauquelin, that the presence of urea occasions common salt to take an octahedral form, although in pure water it crystallizes in cubes, similar to its constituent molecules. The same substance produces an opposite effect upon muriate of ammonia, which crystallizes in octahedrons in pure water; while urea causes it to crystallize in cubes.

A very slight excess or deficiency of base in alum causes it to assume either cubical or octahedral secondary forms; and these forms are so truly secondary, that an octahedral crystal of alum immerged in a solution which is richer in respect to its basis, becomes enveloped with crystalline layers, which causes it to assume at length the form of a cube.

Setting out from these facts, M. Beudant treats the question at full length, and has submitted the crystallization of salts to experiments made under every circumstance that he believed capable of exerting any influence upon it; namely,

1. General and external circumstances; such as heat, weight of the atmosphere, greater or less rapidity of evaporation, bulk of the solution, form of vessels, &c.

2. Mechanical mixtures which foul the solution, whether simply suspended in the state of an incoherent precipitate, or in that of a gelatinous deposit.

3. What he denominates chemical mixtures existing in one and the same solution.

4. Lastly, variations in the proportion of the constituent principles of the crystallized substance.

The circumstances of the first kind do not exercise any other action than what regards the size and perfection of the crystals. The case is even the same as to any small portion of matter which remains in permanent suspension in the liquid; but this cannot be said in respect to precipitates and chemical mixtures.

Crystals formed in the midst of an incoherent precipitate, deposited like mud at the bottom of a liquid, always take up a more or less considerable portion of the molecules of this precipitate, and by this admixture they usually lose all those small additional facets which would otherwise modify their predominant form. Thus the crystalline form acquires greater simplicity when it should apparently become more complicated; at the same time the substances which would otherwise have yielded simple crystals still continue to yield them, and they do not receive any modification.

In a gelatinous deposit, crystals are rarely found in groups, but almost always single, and of a remarkable sharpness and regularity of form; and they do not undergo any variations but those which may result from the chemical action of the substance forming the deposit.

The variations that take place in crystals formed in a chemical mixture, that is to say, in a solution of another substance, are very numerous, even when this substance cannot be united with them. The above-mentioned phenomena are repeated in different forms: common salt crystallized in a solution of borax acquires truncations at the solid angles of its cubes; and alum crystallized in muriatic acid takes a form which M. Beudant has never been able to obtain in any other manner.

If the foreign substance dissolved in the liquid can be united in any proportion whatever with the crystal of another substance that is formed in it, and nevertheless the crystal, by its superior energy, determines the form of the constituent molecule, as we saw last year in the case of sulphate of iron, the matter in the solution will exercise in its turn some influence upon the secondary form of the crystal, and this influence usually consists in simplifying it, and causing the additional facets to disappear.

Thus 30 or 40 per cent. of sulphate of copper may be united to the rhomboidal crystallization of sulphate of iron, but it reduces this sulphate to a pure rhomboid without any truncation either of the angles or of the edges.

A small portion of acetate of copper reduces sulphate of iron to the same simple rhomboidal form, notwithstanding that this form is so disposed to become complicated with additional surfaces.

Other mixtures simplify in a less degree: thus the sulphate of alumine brings that of iron to a rhomboid with the lateral angles only truncated, or what M. Haüy calls his *variété unitaire;* and, whenever this variety of green vitriol is found in the market, where it is very common, we may be sure, according to Mr. Beudant, that it contains alumine.

Lastly, the different proportions between the base and the acid, or, in double salts, between the two bases, produce very sensible effects upon the secondary forms, without altering the primitive form in the least. This has been already exemplified in respect to alum, and M. Beudant has verified it in many other salts.

The author of these researches has made some ingenious applications of these facts to the phenomena afforded by different crystalline mineral substances, upon which direct experiments cannot, in the present state of the science, be made; and he has exhibited some striking analogies: thus natural crystals mixed with foreign substances are in general more simple than others, as is shown in a specimen of axinite, or violet schorl, of Dauphiné, one extremity of which, being mixed with chlorite, is reduced to its primitive form; while the other end, which is pure, is varied by many facets produced by different decrements.

### AUTUMNAL LECTURES.*

### REPORT OF DISEASES.

THE prevalent diseases of the past month have been of a different character from those which may be considered as more proper to the season; and the extent of sickness has been much greater than what is ordinary at this period, which may be accounted for, in a great measure, by the unusually cold north-eastern winds which have prevailed, whilst the sun has excited much power. Acute inflammatory affections of various species, as pleurisy, peritonitis, and bronchitis, have been very common; rheumatism, and acute facial neuralgia, have been often witnessed in the applicants to the Dispensaries. We daily extend the use of the colchicum, accompanied with proper auxiliaries, to those several affections, and with constantly-increased satisfaction.

Some few cases of typhous fever are dispersed about the metropolis; but this cannot be considered as a prevalent form of disease. The cases we have witnessed, seven in number, have been mild, and have all terminated favourably, with very little aid from medicine, after bleeding in the first instance, with emetics, in certain cases, purgatives, &c.

Scarlet fever has been rather common, but not, ordinarily, of a severe character; though one girl, six years of age, died from phrenitis accompanying it: venesection and leeches to the temples had been freely resorted to, without the least advantage. Incessant and most violent vomiting caused everything administered by the mouth to be rejected. Glysters produced stools not of a bad appearance. There was here no affection of the throat; but other children of the same family had scarlet fever with cynanche a few days previously, and the rash on the skin was very well marked in this fatal case.

Small-pox has become much less frequent than in the preceding month, as well as less severe in general. Somewhat, in respect to its mildness, may, probably, be attributed to the increased confidence with which we have lately resorted to blood-letting, in cases attended with highly inflammatory symptoms at the onset.

Cholera morbus has been occasionally witnessed, but not in any instance of an alarming character.

Many cases with symptoms threatening hydrocephalus in children have arisen very obviously from disorders of the intestinal canal, which could often be traced, with great probability, to inordinate use of fruit, unripe or otherwise, of bad quality. Children in these cases often have diarrhœa, and yet a tumid belly. Powerful and repeated doses of calomel and jalap will here commonly bring away a prodigious quantity of dark feculent matter, which is followed by almost instant alleviation of the symptoms. It is too common for such cases to be treated with the chalk mixture and opium, the consequences of which are almost always fatal. Chalk with rhubarb becomes very useful when the irritating matter has been evacuated.

We have succeeded in procuring complete relief in a very severe case of prurigo partibus genitalibus in a man, by the use of an ointment composed of a drachm of powder of white hellebore in an ounce of common cerate, where mercurial, sulphureous, and various other applications had failed to produce any alleviation. The external remedy was accompanied by the internal administration of five grains of Plummer's Pill every night, and two drachms of sulphate of magnesia every morning; but the same medicines had been previously given for a considerable length of time without advantage, whilst other external applications were employed.

### MONTHLY CATALOGUE OF MEDICAL BOOKS.

### METEOROLOGICAL JOURNAL.

By Messrs. WILLIAM HARRIS and Co. 50, Holborn, London.

*From July 20 to August 19, inclusive.*

| Day of Month. | Moon. | Rain gauge | THERM. | | | BAROM. | | DeLuc's Hygro. | | WIND. | | ATMOSPHERIC VARIATION. | | |
|---|---|---|---|---|---|---|---|---|---|---|---|---|---|---|
| July | | | | | | | | | | | | | | |
| 20 | | | 60 | 72 | 58 | 29·62 | 29·82 | 59 | 59 | WSW | W | Fine | | Rain |
| 21 | | '42 | 61 | 71 | 56 | 29·89 | 29·97 | 58 | 57 | W | WNW | Cloud. | Rain | Fine |
| 22 | | | 60 | 70 | 58 | 29·95 | 29·94 | 54 | 54 | WNW | WNW | Fine | | Cloudy |
| 23 | | | 60 | 71 | 57 | 29·95 | 30·00 | 54 | 54 | NW | NW | Cloud. | | |
| 24 | | | 60 | 70 | 61 | 30·00 | 29·93 | 55 | 56 | W | WNW | Cloud. | Fine | Showery |
| 25 | ○ | | 65 | 70 | 55 | 29·88 | 29·94 | 58 | 52 | WNW | NW | Fine | | Showery |
| 26 | | '03 | 61 | 69 | 61 | 30·03 | 30·07 | 53 | 55 | NW | W | Fine | | |
| 27 | | | 65 | 73 | 59 | 30·07 | 30·05 | 57 | 54 | W | W | Cloud. | Fine | |
| 28 | | | 65 | 72 | 63 | 30·04 | 30·06 | 57 | 53 | W | NNE | Fine | | Cloudy |
| 29 | | | 65 | 73 | 62 | 30·09 | 30·09 | 54 | 55 | NNE | WSW | Fine | | |
| 30 | | | 67 | 77 | 60 | 30·08 | 29·91 | 54 | 56 | SSE | SSE | Fine | | Thu.Li.Ra. |
| 31 | | '41 | 66 | 77 | 65 | 29·85 | 29·85 | 56 | 55 | SE | SW | Fine | | Cloudy |
| Aug. | | | | | | | | | | | | | | |
| 1 | ☽ | | 68 | 75 | 58 | 29·82 | 30·00 | 55 | 51 | WSW | WSW | Cloud. | Cloudy | Cloudy |
| 2 | | | 61 | 73 | 62 | 30·09 | 30·11 | 53 | 52 | WSW | SW | Fine | | Cloudy |
| 3 | | | 65 | 75 | 70 | 30·00 | 29·84 | 56 | 57 | SW | SW | Cloud. | | |
| 4 | | | 68 | 76 | 58 | 29·76 | 29·83 | 59 | 55 | WSW | WSW | Rain | Showery | Cloudy |
| 5 | | '07 | 62 | 73 | 58 | 29·84 | 29·85 | 54 | 55 | W | SW | Fine | | Rain |
| 6 | | '11 | 62 | 68 | 55 | 29·70 | 29·64 | 59 | 55 | SW | SW | Rain | | Cloudy |
| 7 | | '12 | 58 | 66 | 55 | 29·75 | 30·00 | 55 | 53 | WNW | W | Fine | Showery | Fine |
| 8 | ● | | 59 | 62 | 58 | 30·00 | 29·83 | 55 | 53 | WSW | WSW | Fine | | Cloudy |
| 9 | | | 61 | 68 | 57 | 29·97 | 30·18 | 55 | 53 | W | WNW | Fine | | |
| 10 | | | 60 | 69 | 60 | 30·27 | 30·37 | 55 | 53 | SW | WSW | Fine | | |
| 11 | | | 62 | 70 | 59 | 30·27 | 30·19 | 56 | 54 | W | W | Fine | | |
| 12 | | | 61 | 71 | 58 | 30·18 | 30·13 | 54 | 53 | N | NNE | Fine | | |
| 13 | | | 60 | 66 | 59 | 30·11 | 30·03 | 52 | 54 | NNE | ENE | Cloud. | Fine | Fine |
| 14 | | | 65 | 72 | 59 | 29·98 | 29·92 | 50 | 52 | WNW | WNW | Fine | | |
| 15 | | | 61 | 70 | 62 | 29·90 | 29·81 | 52 | 56 | WSW | WSW | Fine | | |
| 16 | | | 65 | 73 | 63 | 29·81 | 29·88 | 58 | 57 | SW | WSW | Cloud. | Fine | Cloudy |
| 17 | ☾ | | 68 | 72 | 59 | 29·80 | 29·86 | 58 | 54 | SW | NNW | Cloud. | Fine | |
| 18 | | | 61 | 68 | 57 | 29·81 | 29·79 | 52 | 49 | N | WNW | Cloud. | Fi. Lightn. | |
| 19 | | | 59 | 63 | 50 | 29.73 | 29·82 | 52 | 53 | NE | WNW | Fine | Rain | Fine |

The quantity of rain fallen in July, is 2 inches and 51-100ths.

*** *Some of our Subscribers have, it appears, suffered a little inconvenience from the delay in the publication of the* INDEX *to the last Volume: we shall have measures taken in future to obviate this, by having it prepared so early that it may be attached to the last Number of each respective Volume.*

